# Consensus Guidance for Screening, Classification, and Treatment of Retinopathy Associated with Incontinentia Pigmenti

**DOI:** 10.1016/j.xops.2026.101293

**Published:** 2026-06-19

**Authors:** Jade Y. Moon, Becky Abbott, Audina Berrocal, Connie Chen, Xi Chen, Philip Ferrone, Mary Fete, Morton F. Goldberg, Dorothy K. Grange, Ian Han, M. Elizabeth Hartnett, Robert H. Henderson, Eric Nudleman, Sengul Ozdek, Richard N. Sather, Nicole Somani, Reid Wilson, Sandra R. Montezuma, J. Peter Campbell

**Affiliations:** 1 Department of Ophthalmology and Visual Neurosciences, University of Minnesota, Minneapolis, Minnesota; 2 National Foundation of Ectodermal Dysplasias, Fairview Heights, Illinois; 3 Bascom Palmer Eye Institute, University of Miami, Miami, Florida; 4 Department of Ophthalmology, Virginia Mason Medical Center, Seattle, Washington; 5 Department of Ophthalmology, Duke Eye Center, Durham, North Carolina; 6 Department of Ophthalmology, Vitreoretinal Consultants of New York, Great Neck, New York; 7 Department of Ophthalmology and Visual Sciences, University of Iowa, Iowa City, Iowa; 8 Department of Ophthalmology, Wilmer Eye Institute at Johns Hopkins University School of Medicine, Baltimore, Maryland; 9 Division of Genetics and Genomic Medicine, Department of Pediatrics, Washington University School of Medicine, St. Louis, Missouri; 10 Department of Ophthalmology, University of Iowa, Iowa City, Iowa; 11 Byers Eye Institute, Stanford University, Palo Alto, California; 12 Great Ormond Street Hospital, London, United Kingdom; 13 Shiley Eye Institute, University of California San Diego, San Diego, La Jolla, California; 14 Department of Ophthalmology, Gazi University School of Medicine, Ankara, Turkey; 15 Casey Eye Institute, Oregon Health and Science University, Portland, Oregon

**Keywords:** Incontinentia pigmenti, Pediatric retina, Vascular retinopathy, Screening guidelines, Blindness

## Abstract

**Objective:**

Incontinentia pigmenti (IP) is a rare genetic condition that can lead to blindness. Clinical practice for screening for the ocular complications of IP is variable. Current literature lacks consensus for the screening, classification, and treatment of IP-related eye disease despite substantial risk of retinal detachment (RD) and blindness. This report summarizes the state of knowledge and establishes expert guidance for screening, classification, and treatment of IP-related eye disease from the International Conference on Incontinentia Pigmenti (ICIP) sponsored by the National Foundation for Ectodermal Dysplasias in February 2025.

**Design:**

Literature review.

**Subjects:**

Images from authors' patients with a diagnosis of IP were included in this study.

**Methods:**

We review the current literature on screening and treatment of retinal complications of IP and provide guidance on disease screening, classification, and management from the ICIP in 2025.

**Main Outcome Measures:**

Screening, treatment, and surveillance options were reviewed.

**Results:**

The ICIP established guidance to support parents and ophthalmologists in appropriate screening and treatment of infants diagnosed with IP. Due to the high prevalence of retinal involvement in IP, infants diagnosed with IP should receive an early, comprehensive retinal examination, ideally with widefield fluorescein angiography. Like treatment considerations for other pediatric diseases with retinal ischemia, treatment concentrates acutely on regression of neovascularization and strategies to reduce incidence of RDs later in life.

**Conclusions:**

Incontinentia pigmenti is a rare but complex multisystem disorder with the potential for serious retinal complications. Careful ophthalmic screening and surveillance are crucial to optimize patients' visual outcomes.

**Financial Disclosure(s):**

Proprietary or commercial disclosure may be found in the Footnotes and Disclosures at the end of this article.

### Background

Incontinentia pigmenti (IP) is a rare ectodermal dysplasia with an estimated prevalence of 1.2/100 000.^1^ Initially described by Swiss dermatologist Bruno Bloch in 1926 and American dermatologist Marion Sulzberger in 1928, and initially known as Bloch–Sulzberger syndrome, the disease was later termed IP. “Incontinentia” refers to the incontinence or migration of melanin from the basal layer of the epidermis to the dermis, and “pigmenti” also refers to the hyperpigmented swirls and streaks seen clinically in the later stages of the disease. Incontinentia pigmenti is caused by pathogenic variants in the *IKBKG* (inhibitor of nuclear factor kappa-b kinase, regulatory subunit gamma) gene, previously referred to as *NEMO* (NF-kappa-B essential modulator), first described in 2000.^2^^,^^3^ Approximately 70% to 80% of IP patients have a causative 11.7 kb deletion in *IKBKG*, with about 20% of patients having no identifiable variant.^4^ Further, it is estimated that about 75% of cases are sporadic.^5^

Mutation in *IKBKG* disrupts the ability of cells to respond to signals that prevent cell death, leading to the progressive skin and multisystem symptoms characteristics of IP.^6^^,^^7^ *IKBKG*-deficient keratinocytes cannot activate nuclear factor kappa-b in response to tumor necrosis factor, making them vulnerable to tumor necrosis factor–induced cell death and associated inflammation.^8^ It is believed that this inflammation leads to vaso-occlusion in small capillary beds in affected organ systems throughout the body, including in the cerebral vasculature, but best visualized in the retina.^9^ Furthermore, Protein Arginine Methyltransferase 1 and 5 are most highly expressed in keratinocytes and are activated by the nuclear factor kappa-b pathway, critical for normal development of the epidermis.^10^ Downregulation of this process can disrupt cutaneous stratification and result in ectodermal dysplasia, as seen in IP.

Pathogenic variants in *IKBKG* primarily impact females due to the X-linked dominant inheritance pattern. Females have a normal copy of IKBKG on their other X chromosome that can partially compensate, whereas pathogenic variants are generally lethal in males. However, there are rare cases of mosaicism, where only a portion of an individual's cells carry the pathogenic variant in *IKBKG* responsible for the disease, while allowing other cells to function normally, or cases of hypomorphic mutations, where there is a partial loss-of-function, that allow some males to present with IP signs and symptoms.^11^^,^^12^ In addition, males with Klinefelter syndrome, a genetic condition associated with an extra X chromosome (47, XXY), can present with IP without lethality when 1 X chromosome carries a pathogenic variant.^6^^,^^11^

### Skin and Other Systemic Findings in IP

Incontinentia pigmenti is most diagnosed through recognition of a congenital or perinatal rash. The distinct skin lesions in IP follow a specific pattern that evolves through 4 stages, reflecting underlying changes in cell and immune dynamics: vesicular (blistering), verrucous (wart-like growths), hyperpigmentation, and finally, hypopigmentation (light patches or scarring) (Fig 1).^13^ Accordingly, histopathological findings vary based on the stage of the lesions. In the first phase, epidermal hyperplasia and perivascular infiltrates of eosinophils, neutrophils, and rarely basophils are observed with large dyskeratotic cells. The verrucous phase presents with acanthosis, hyperkeratosis, and papillomatosis. The hyperpigmentation phase is described by prominent pigmentary incontinence. The atrophic phase is characterized by the absence of pigment in the epidermis and loss of eccrine glands.^14^^,^^15^

**Figure 1:**
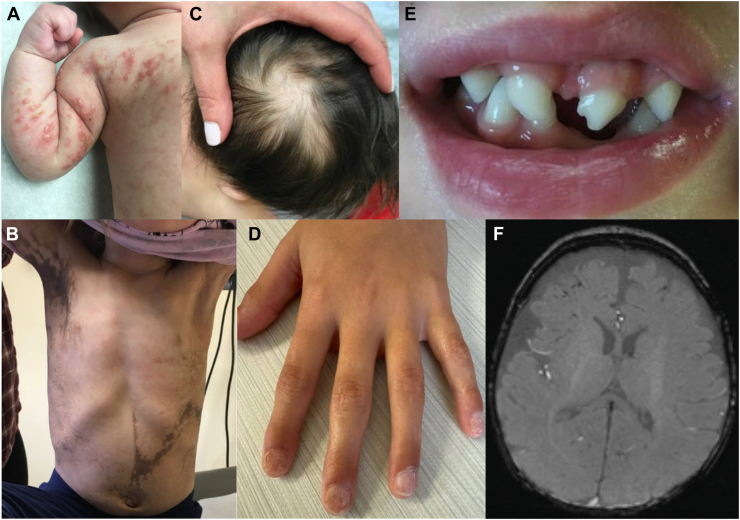
Systemic findings of incontinentia pigmenti. Skin findings include blistering **(A)** and hyperpigmentation **(B)**. Other findings include alopecia **(C)**, dystrophic nails **(D)**, and abnormally shaped teeth **(E)**. Magnetic resonance imaging of the brain **(F)** shows hyperintense lesions in the bilateral frontal subcortical region, which is concerning for subacute to old microhemorrhages that can be associated with incontinentia pigmenti.

Beyond dermatologic findings, IP can affect multiple organ systems, including the eyes, CNS, teeth, and hair (Fig 1). Many of these findings are believed to be secondary to overexpression of proinflammatory cytokines and their sequelae.^9^ Neurological symptoms are highly variable and can be severe, with approximately 30% of individuals experiencing intellectual disability, seizures, or motor delays.^16^^,^^17^ Dental abnormalities are also common, including missing or peg-shaped teeth.^18^ Additional complications may include jaw malocclusion, conical adult teeth, hypodontia, and delayed tooth eruption.^19^

### Ocular Manifestations of IP

Ocular complications in IP can present a substantial risk of vision impairment and are associated with characteristic retinal vascular anomalies. Historically, about a third of patients with IP were thought to have eye diseases.^20^, ^21^, ^22^ However, recent literature has suggested that this figure may be an underestimation, and most recently, Danford et al^23^ have suggested that greater than 70% of babies show angiographic signs of retinal involvement. These include ischemia, neovascularization, and subsequent traction, which could compromise the retinal structure and stability and increase the likelihood of RD—a leading cause of irreversible vision loss in IP if unmanaged.^20^

While the pathophysiology of IP-associated retinopathy is not completely understood, it is believed that retinopathy is due to vaso-occlusive ischemia in the setting of inflammation, possibly with an associated eosinophilic vasculitis.^24^^,^^25^ Retinal detachment (RD) in IP can either be tractional, due to forces exerted by neovascular membranes and fibrotic tissue, or rhegmatogenous, from atrophic holes at the border of perfused and nonperfused retina.^20^^,^^21^ The retinopathy in IP is further characterized by potential spontaneous regression and its bimodal age distribution of RDs, with most tractional RDs occurring in early childhood and most rhegmatogenous RDs occurring in adulthood.^20^ Although vision loss most commonly is a result of RD, less frequently, it can be due to vitreous hemorrhage, retinal arterial occlusion, ischemic optic neuropathy, or occipital lobe infarction.^20^ Secondary manifestations, such as strabismus, nystagmus, optic atrophy, and occasionally microphthalmia, are likely attributable to disrupted retinal development and altered blood vessel formation associated with pathogenic *IKBKG* gene variants.^22^

### Diagnosis of IP

Diagnosis of IP is primarily based on clinical findings.^26^ Confirmatory genetic testing is not required. Diagnostic criteria can be divided into major criteria and minor criteria. Major criteria include all the stages of hallmark skin findings. Minor criteria include all other systemic manifestations, including retinopathy. In patients without a family history of IP in first-degree relatives, patients are diagnosed with IP if the patient has a pathogenic variant in the IKBKG gene as well as any single major or minor criterion or meets ≥2 major criteria or 1 major and ≥1 minor criterion without genetic testing data. For those with a family history of IP in first-degree relatives, patients must demonstrate any single major criterion or ≥2 minor criteria for a diagnosis of IP. In cases where the ophthalmologist is the primary provider taking care of these patients or making the diagnosis, a prompt referral to the patient's pediatrician should be made for coordination for further interdisciplinary care. Genetic testing can be considered, though not necessary.

### Current Screening and Staging Guidelines for IP-Associated Retinopathy

Previously proposed vision screening guidelines for IP emphasize the critical need for early and ongoing ophthalmic evaluations, given the high risk of vision-threatening retinal complications associated with the disease. In contrast to retinopathy of prematurity (ROP), where evidence-based screening criteria are explicitly guided by gestational age and birth weight, the screening for IP lacks clear recommendations in the literature and has historically been guided by systemic findings in addition to clinical judgment. For instance, the presence of cutaneous lesions is a hallmark indicator for ocular screening. However, it is important to note that while skin lesions are present at birth or develop within 2 weeks of birth in 90% of patients, it is not always present and may not necessarily be the best indicator of eye disease.^14^ Additionally, as 75% of cases are sporadic and present without family history of IP, there can be a delay in diagnosis or even misdiagnosis. A recent study indicates that patients with retinal findings were diagnosed at a median age of 19 days.^27^ However, other studies suggest that the median age of diagnosis may be later, approximately 3.1 months, due to delayed identification of systemic features. Further, unlike ROP, where once the retina is fully vascularized, the sight-threatening risks subside, patients with IP-associated retinopathy require lifelong monitoring due to the condition's bimodal age distribution of associated risk for developing RD.^20^

Despite the complexity of the disease and its vision-threatening complications, there is little consensus regarding the optimal timing and frequency of ophthalmic examinations, although many prior screening guidelines have been proposed.^20^^,^^22^^,^^27^, ^28^, ^29^ These guidelines generally agree on the importance of early and frequent screening as well as the need for longitudinal follow-up, but the exact timing and interval of exams differ (Table 1).

**Table 1:** Summary of Screening, Staging, and Treatment Guidelines in Key Publications

Paper	Year of Publication	Study Design	Screening Recommendations	Staging Suggestions	Treatment Suggestions
Holmstrom et al^22^	2000	Observational cohort study of 30 patients.	• Screening examination as soon as possible and at least monthly for 3–4 mo • Afterward, continue examinations every 3 mo for 1 yr then twice yearly until age 3 • If no disease is detected by age 3, follow-up examinations can be stopped	• Grade 1: minor RPE changes • Grade 2: vascularized or nonvascularized temporal ridge, with or without traction • Grade 3: RD, retrolental membrane or mass	Cryo or laser treatment is recommended when early signs of progression of retinal disease are present. Treatment of avascular retina can be considered, although success rate varies.
Chen et al^20^	2015	Observational cohort study of patients with IP that included 50 eyes of 25 female patients. Duration of follow-up ranged from 6 mo to 22.8 yrs.	• Screening examination as soon as possible, preferably under general anesthesia and with FA • Follow-up determined by severity of retinopathy • Follow-up interval can be decreased to every 6–12 mo if the disease becomes stabilized by age 2 • Patients with evidence of nonperfusion or proliferative retinopathy should be monitored throughout adulthood	Not explicitly discussed	Laser photocoagulation or cryotherapy of nonperfused retina should be limited to patients who show documented progression of neovascularization, progression of vitreous traction, or vitreous hemorrhage at successive visits
Huang et al^27^	2017	Literature review of 42 peer-reviewed papers containing 60 unique patient cases of 100 affected eyes with documentation of the diagnosis and treatment	• Initial retinal screening examination should be as early as possible with EUA and FA or UWFP and FA • If no retinopathy, repeat examination every 3–6 mo for 1 yr then semiannual until age 3 then annual for life • If grade 1 disease, consider treatment with photocoagulation with follow-up examination with UWFP and FA in 6–8 wk. If untreated, follow-up every 2 wk for 3 mo and monthly for 6 mo and repeat UWFP and FA at 3 mo. • If grade 2 disease, treat with laser photocoagulation or cryotherapy and follow-up with UWFP and FA in 6–8 wk. Could consider intravitreal anti-VEGF as sole or adjunctive treatment. • If grade 3 disease, vitreoretinal surgery • For all patients, once retinal disease is stabilized, repeat examination every 3 mo until 1 yr of age and repeat FA at least once during first year. Afterward, semiannual office visits and UWFP and FA once during second year. Then, follow-up semiannually until age 5 then annually afterward	• Grade 0: normal retina without retinopathy • Grade 1: incomplete vascularization of the peripheral retina • Grade 2: retinal neovascularization or retinal hemorrhage • Grade 3: tractional or rhegmatogenous RD • Grade 4: chronic complete RD and development of retrolental fibrovascular membrane/mass	See screening recommendations
O’Doherty et al^28^	2017	Observational case series of 11 patients and literature review of 15 papers with ocular findings of IP	• Screening examination should be performed immediately after birth • If screening examination is normal, biannual follow-up • If screening examination is abnormal, FA should be performed as soon as possible with repeat examinations every 2 wk for the first 3 mo, then monthly for 6 mo then quarterly for 1 yr	Not explicitly discussed	Early treatment with peripheral retinal photocoagulation to areas of peripheral ischemia and neovascularization is recommended. Normal-appearing retina requires only routine surveillance.
Michel et al^26^	2020	Retrospective study of 38 eyes of 19 patients with diagnosis of incontinentia pigmenti. Duration of follow-up ranged from 3.0 yrs to 9.8 yrs.	• Initial examination should be performed within 48 h of diagnosis. • If normal examination, repeat examination at months 1, 2, 3, 6, 9, 12, 18, and 24 followed by annual examinations until age 15. • If there are signs of vasculopathy, recommend an urgent EUA within 5 d. If vasculopathy is confirmed during EUA, recommend treatment followed by postintervention screening at months 1, 2, and 3. If there are no new lesions, can resume the standard screening. If new lesions are found, repeat an urgent EUA and treatment as needed.	Not explicitly discussed	Photocoagulation therapy is recommended to areas of nonperfused retina.
Danford et al^23^	2022	Multi-institutional retrospective case series of 40 patients	• A screening examination with FA should be strongly considered as soon as IP is diagnosed • Untreated eyes with ischemia should be monitored with serial EUA’s every 1–2 mo until the child is stable then every 4–12 mo	Based on ophthalmoscopic findings and FA images • Grade 0: no disease • Grade 1: vascular abnormalities without leakage • Grade 2: leakage or neovascularization • Grade 3: retinal detachment	Treatment considerations include laser photocoagulation, intravitreal anti-VEGF injections, and pars plana vitrectomy. However, specific indications are not explicitly discussed.

EUA = examination under anesthesia; FA = fluorescein angiography; IP = incontinentia pigmenti; RD = retinal detachment; RPE = retinal pigment epithelium; UWFP = ultra-widefield fundus photography.

Similarly, controversy remains regarding severity staging for ophthalmic complications in IP. While severity of retinopathy is generally determined by degree of ischemia and presence of neovascularization on ophthalmoscopic examination similar to the staging system for ROP, many groups have proposed unique grading schema of the severity of retinopathy.^22^^,^^28^ Most recently, Danford et al^23^ have suggested that reliance on ophthalmoscopy may not be sufficient to detect the clinical spectrum of retinopathy, but rather evaluation with fluorescein angiography (FA) may be necessary.

### Current Treatment Guidelines

The primary goal of ophthalmic treatment is to decrease the likelihood of vision loss by preventing progression to proliferative retinopathy and its associated complications (e.g., vitreous hemorrhage, neovascular glaucoma) and to prevent or treat tractional, rhegmatogenous, or combined tractional-rhegmatogenous RD.^24^ However, studies evaluating the management of retinal complications of IP are sparse, and in many ways, current treatment for the retinal vascular manifestation of IP relies on evidence for treatment options for ROP.^30^ As such, previously reported management options include observation for mild disease, photocoagulation, anti-VEGF therapy for proliferative disease, and surgery in cases of RD.

While all clinicians agree that there are cases that benefit from therapeutic intervention, the lack of high quality evidence leads to variations in practice patterns regarding the timing of intervention and balancing the relative risk of observation versus treatment.^20^ For example, there are reports of RDs developing after laser treatment, possibly secondary to tissue contraction in regressing neovascularization; such a complication may occur in all proliferative retinopathies, but in IP, the absence of level I evidence for treatment benefit requires clinical judgment and due caution. Perhaps unique to IP are rare cases of arterial and venous occlusion after laser treatment, which could be indicative of the natural history of disease, or secondary to laser-induced inflammation and vascular occlusion.^31^ At the same time, similar to other retinopathies, neovascularization can regress spontaneously even without treatment, raising questions as to the short term disease modifying effect of the therapeutic interventions.^20^

With the recent adoption of the use of intravitreal injections of anti-VEGF agents, there has been a growing consideration of use of anti-VEGF therapy in conjunction with laser therapy for the management of many ischemic retinal diseases, including IP.^32^ However, current evidence to support the use of anti-VEGF injections in IP is limited to uncontrolled case reports.^33^, ^34^, ^35^

Once patients develop an RD, vitreoretinal surgery may be necessary. The surgical approach is similar to that of other pediatric proliferative retinopathies. To date, there is a paucity of data on success rates of retinal surgery in eyes with IP. It is also important to note that IP can result in tractional, rhegmatogenous, or combined tractional and rhegmatogenous RDs, and eyes with rhegmatogenous RD overall had better surgical outcomes compared to those with tractional RD.^20^ A previous observational cohort study has suggested that tractional RDs tend to present early in life, whereas rhegmatogenous RDs tend to present later in life due to holes in atrophic, avascular retina.^20^^,^^36^ These 2 different modes of presentation, requiring different surgical techniques, further the challenge in understanding the surgical outcomes in this rare condition. These gaps in knowledge emphasize the need for continued collaboration and building of multicenter databases to enhance our understanding of the disease complications and potential outcomes after treatment.

### Late Sequelae of IP

Large-scale studies investigating the natural history of IP-associated retinopathy are also scant. However, case reports and case series suggest that IP exhibit a spectrum of retinal abnormalities long term:

• Peripheral avascular retina. There may be spontaneous regression of neovascularization.^20^ Similar to ROP, there is increasing awareness of the potential long-term risks of untreated peripheral avascular retina, which is the rationale some clinicians use to perform laser treatment to avascular retina less for disease modification and more for (presumed) reduction of long-term risk of RD.
• Vascular remodeling. Both peripheral and macular vascular remodeling have been observed.^37^, ^38^, ^39^, ^40^, ^41^ Observed changes include decreased vascular density, increased foveal avascular zone, and abnormal vascular loops.
• Retinal thinning. Studies have demonstrated atrophic thinning of both inner and outer retina regardless of treatment.^41^^,^^42^
• Tractional RD and retinoschisis. Tractional retinoschisis has been described in the literature.^37^^,^^43^ Similar to other pediatric retinopathies, tractional RDs are typically observed early on in life, though there are reports of late presentation of tractional RDs years after initial presentation, both later in childhood or in adulthood (Fig 2).^20^^,^^44^

**Figure 2:**
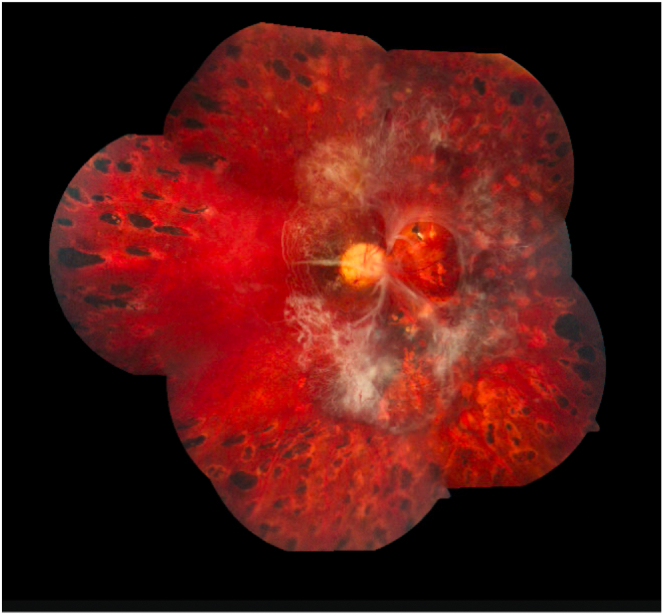
An adult patient with IP who had prior laser treatment presented with a tractional retinal detachment. This patient initially presented after receiving laser photocoagulation at an outside clinic. At the time of presentation, patient had stage 2 IP-associated retinopathy, and additional laser photocoagulation was performed to areas of avascular retina. IP = incontinentia pigmenti.
• Rhegmatogenous RD. In 1 case series, eyes with rhegmatogenous RD developed atrophic holes at the border of perfused and nonperfused retina. In this study, all eyes with rhegmatogenous RDs developed after the age of 14.^20^

### The Role of Modern Retinal Imaging in Management of IP-Associated Retinopathy

Over the past few decades, advances in retinal imaging have revolutionized our approach to diagnosis and monitoring of IP-associated retinopathy. One of the most important advances was the development of FA. First reported in 1961, FA allowed clinicians to detect subtle vascular changes in the human retina, and subsequent iterations have allowed higher resolution images and wider field of view.^45^ Due to its ability to identify areas of vascular nonperfusion and regions of other vascular abnormalities, FA is especially useful in the diagnosis and monitoring of ischemic retinopathies, including IP-associated retinopathy. Common widefield FA (WFFA) findings in eyes with nonproliferative IP-associated retinopathy include peripheral retinal ischemia, vascular tortuosity, vascular loops, and neovascularization.^46^ Widefield FA can also be useful for guiding management by identifying areas of ischemia and is often utilized during examinations under anesthesia (EUAs) to identify occult areas of neovascularization and leakage that may later progress to RD.^47^ Indeed, Danford et al^23^ suggested that FA is more sensitive than ophthalmoscopic examination in detection of IP-associated retinopathy (Fig 3). Additionally, foveal hypoplasia has been well-described in the literature in patients with IP, and FA is useful for identifying macular vascular abnormalities in IP, which are often associated with reduced vision.^41^^,^^48^^,^^49^

**Figure 3:**
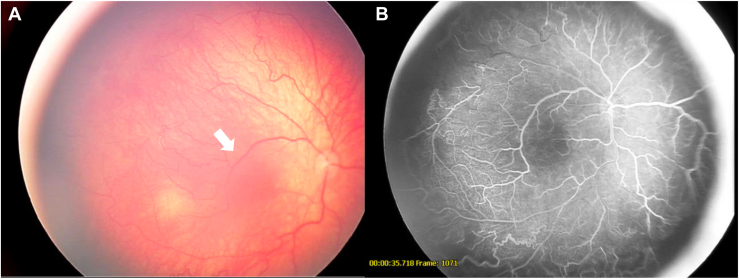
Color fundus photography **(A)** and fluorescein angiography **(B)** of stage 1 IP-associated retinopathy highlight the need for fluorescein angiography for detailed characterization of vascular abnormalities in IP. Fundus photography demonstrates a large vessel that crosses the macula from the superior arcade to the inferior arcade territory (white arrow). Fluorescein angiography additionally highlights peripheral vascular abnormalities with corkscrew retinal tortuosity, peripheral arterial-venous communications, mild early leakage, and peripheral capillary dropout. IP = incontinentia pigmenti.

The development of OCT also marked a pivotal milestone in the field of ophthalmology.^50^ OCT enabled clinicians to examine the structural integrity of the retina in a noninvasive manner via cross-sectional images. In IP, OCT has been used to identify inner retinal thinning and irregularities in the outer plexiform layer, likely due to patchy multifocal posterior nonperfusion (Fig 4A–G).^37^^,^^51^ Other OCT findings include early loss of layer integrity, similar to other occlusive retinal vascular disease, followed by later thinning of the inner retina, and abnormal inner foveal structure.^41^^,^^52^

**Figure 4:**
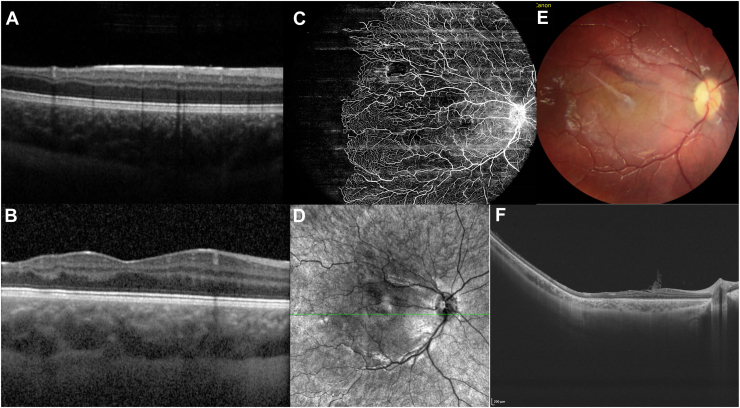
(**A** and **B**), Spectral domain OCT findings of a 5-year-old patient with stage 2 IP show significant inner retina thinning **(A)** and irregularities of the outer plexiform layer (**B**). (**C**), OCT angiography of a different 5-year-old patient with stage 2 IP demonstrates peripheral avascular retina and capillary nonperfusion. (**D**, **E**, and **F**), Fundus image **(E)** and widefield OCT **(F)** with en face image **(D)** of a third 5-year-old patient demonstrate macular tractional membrane and temporal retinal thinning. IP = incontinentia pigmenti.

In recent years, OCT angiography (OCTA) has arisen as a safer, noninvasive alternative to FA. Compared to FA, OCTA can provide a more detailed view of the retinal vasculature (Fig 4C). Further, OCTA can be more sensitive than FA in identifying vascular abnormalities.^31^ In addition to peripheral avascular retina, neovascularization, and capillary drop out, previous reports of OCTA images of patients with IP have demonstrated macular vascular changes, including decreased vascular density of both the superficial and deep capillary plexus, decreased capillary flow, flow loss in the areas of inner and outer retinal thinning suggesting sequential ischemic events, enlarged foveal avascular zone, and abnormal vascular loops.

Retinal imaging is a powerful complement to the clinical examination, guiding both diagnosis and management. As retinal imaging continues to evolve, permitting a wider and more detailed visualization of the peripheral retina, our understanding of the disease pathophysiology, as well as disease progression and visual potential, may also evolve.

## The International Conference on Incontinentia Pigmenti—Expert Guidance on Screening, Disease Classification, and Management in IP

In February 2025, the National Foundation for Ectodermal Dysplasias and the Oregon Health Sciences University cosponsored an International Conference on Incontinentia Pigmenti (ICIP). The ICIP was organized to bring together experts in multiple fields, including genetics, neurology, dentistry, dermatology, and ophthalmology, to develop a shared understanding of IP, leading to improved interdisciplinary collaboration. A substantial portion of the ICIP was devoted to reviewing the existing literature on ophthalmic manifestations in IP and developing expert guidance in areas of controversy, as highlighted above. Recognizing the current lack of an agreed-upon screening, grading, and treatment schema for IP-associated retinopathy, the ICIP participants proposed a new standardized classification based on FA that outlines the key features of IP-associated retinopathy to guide the clinical decision-making process.^31^ This updated recommendation carefully considered the individual experiences of expert physicians, many of whom have authored the existing guidelines in the literature, as well as mindful allocation of limited resources. Overall, these guidelines highlight the importance of early ophthalmic examination with possible early intervention.

This review was performed in accordance with the Health Insurance Portability and Accountability Act of 1996 and the Declaration of Helsinki. The Institutional Review Board at the University of Minnesota approved the study protocol. A written informed consent was obtained from all patients whose images were included.

### Screening for IP-Related Ophthalmic Complications

International Conference on Incontinentia Pigmenti participants proposed the following screening process for newly diagnosed or suspected patients with IP. Patients with suspected or confirmed IP should be urgently referred to a pediatric ophthalmologist or retina specialist for a dilated ophthalmoscopic examination with scleral depression within 1 month of diagnosis. If bedside dilated ophthalmoscopic examination is not possible, EUA may be required. If retinal abnormalities are present during the initial examination, WFFA and treatment should follow as soon as possible, ideally within 1 month. If the initial ophthalmoscopic examination is normal, a screening WFFA should be performed within 3 months, given that many infants will have evidence of disease on WFFA that is not visible clinically.

### Staging of IP-Associated Retinopathy

**Figure 5:**
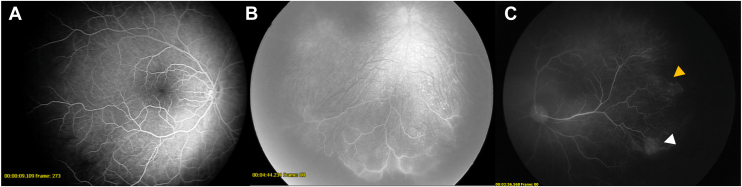
Classification of IP-associated retinopathy based on WFFA. (**A**), Patient with stage 0 retinopathy with normal fluorescein angiography. (**B**), Patient with stage 1 retinopathy with significant areas of leakage and nonperfusion inferiorly. (**C**), Patient with stage 2 retinopathy with significantly delayed temporal filling with abnormal temporal telangiectatic vessels (yellow arrowhead), late leakage, inferior extraretinal neovascularization (white arrowhead), and peripheral nonperfusion. Stage 3 represents retinal detachment. WFFA = widefield FA; IP = incontinentia pigmenti.

The ICIP participants proposed the following consensus grading scheme for IP-associated retinopathy based on dilated ophthalmoscopic examination and WFFA (Fig 5):

• **Stage 0**: no retinal disease. We define normal peripheral nonperfusion as 1.5 disc diameter or less temporally and 1.0 disc diameters or less nasally.^53^
• **Stage 1**: avascular retina or retinal vascular abnormalities, including arterial occlusion, with or without leakage
• **Stage 2**: extraretinal neovascularization without RD
• **Stage 3**: RD, with or without foveal involvement

### Treatment and Monitoring Guidelines for IP-Associated Retinopathy

At the present time, there is no level 1, 2, or 3 evidence regarding the safety and efficacy of these therapeutic interventions, and level 4 evidence is also limited. The following treatment recommendations reflect the consensus of the ICIP participants developed to provide general guidelines for patients, families, and pediatricians for acceptable current practice based on available knowledge. The authors acknowledge that given the low prevalence of disease, it may never be possible to develop higher quality evidence, and that this practice patterns may evolve over time with new information.

• **Stage 0** (no retinal disease): observation is recommended. To the authors' knowledge, there have been no cases of disease progression following a normal WFFA at baseline. Nonetheless, given the rarity of the condition and the fact that image quality can be challenging, especially in small eyes with low doses of fluorescein, it may be prudent to repeat WFFA within 6 months if there is any concern. Thereafter, an annual dilated fundus examination (DFE) should be performed with consideration of additional WFFA at the discretion of the physician.
• **Stage 1** (avascular retina or retinal vascular abnormalities with or without leakage): there are insufficient data to support the effectiveness of either laser treatment or anti-VEGF therapy as disease modifying interventions. However, some of the authors consider laser photocoagulation to avascular areas in these eyes given long-term risk of avascular retina, including the development of neovascular complications that may be missed in infants, and if there is a risk of a patient being lost to follow-up. If the physician chooses to observe without treatment, a DFE should be performed at least every 3 months, and WFFA should be performed at least every 6 months for the first 1-2 years. If there is evidence of stability of disease, follow-up examinations can be spaced out to annual DFE with or without WFFA at the discretion of physician. If the physician chooses to treat with photocoagulation, a subsequent follow-up examination should occur within 3 months (clinic or EUA at physician discretion) and repeat WFFA within 6 months. Based on the literature and authors' knowledge, there are rare cases of disease progression of retinopathy despite treatment with laser photocoagulation.^20^ However, some suggest that early and aggressive intervention may prevent disease progression.^27^ Thus, longitudinal follow-up is critical, and patients and families should be carefully counseled. If there is evidence of active disease on repeat WFFA, further treatment should be considered with follow-up interval determined by the updated stage of disease as defined above. Should there be stabilization of disease on the follow-up WFFA without additional areas of vascular abnormalities or avascular retina, an annual DFE should be performed with consideration of additional WFFA at the discretion of the physician.
• **Stage 2** (extraretinal neovascularization): in the presence of neovascularization, most authors would recommend laser photocoagulation to areas of avascular retina, with some also recommending anti-VEGF therapy. Although spontaneous regression of neovascularization can occur in IP, laser may reduce the risk of both early progression to tractional RD, and later progression to rhegmatogenous RD. Closer follow-up is recommended given more advanced disease, with repeat examination within 2 months (clinic or EUA at physician discretion) and WFFA within 4 months. If there is evidence of active disease on repeat WFFA, further treatment should be considered with follow-up interval determined by the updated stage of disease as defined above. Should there be stabilization of disease on the follow-up WFFA without additional areas of vascular abnormalities or avascular retina, an annual DFE should be performed with consideration of additional WFFA at the discretion of the physician.
• **Stage 3** (RD, with or without foveal involvement): pediatric vitreoretinal surgical consultation and potential intervention at the discretion of the pediatric vitreoretinal surgeon is indicated as soon as possible.

**Figure 6:**
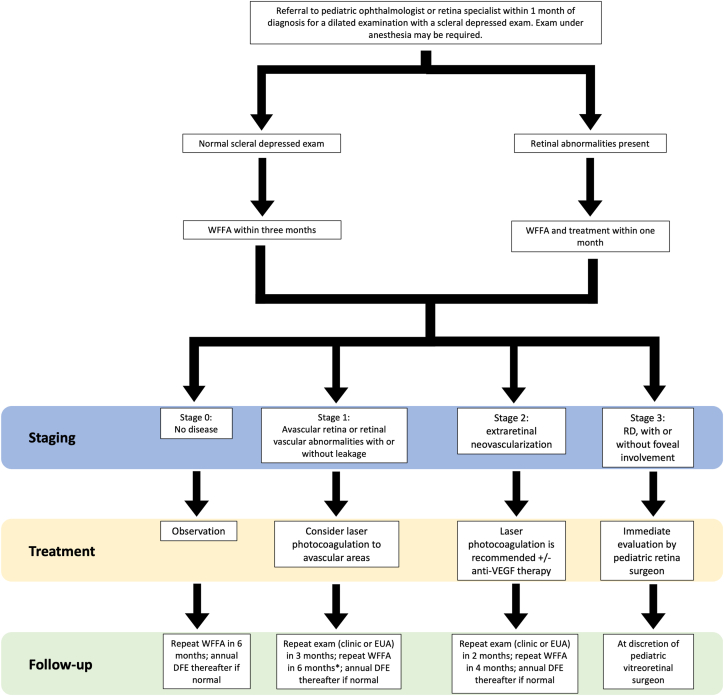
Schematic of screening, treatment, and follow-up recommendations. Examinations may be performed in clinic or via EUA. ∗If clinician chooses to observe stage 1 disease, this recommended screening interval of clinic examination at least every 3 months and WFFA at least every 6 months should be continued for 1 to 2 years at the discretion of the physician. If stability is demonstrated, examinations can be spaced out to annual DFEs with or without WFFA. DFE = dilated fundus examination; EUA = examination under anesthesia; RD = retinal detachment; WFFA = widefield fluorescein angiography.

Proposed screening and treatment protocol is detailed in Figure 6.

## Future Directions

With further advances in sequencing technologies, our diagnostic capabilities to genotype and classify mutations in the *IKBKG* gene will improve. In turn, clinicians will be able to perform a more in-depth analysis of genotype-phenotype correlation. This could translate not only to a better understanding and prognostication of the disease but also to improved treatment decisions.

While no gene therapy is currently available for IP, the advances in gene-editing technologies such as clustered regularly interspaced short palindromic repeats offer promising avenues for addressing the underlying genetic defects. In one study, clustered regularly interspaced short palindromic repeats associated protein 9 was utilized to create an *IKBKG* (*NEMO*) gene knockout in induced pluripotent stem cells.^54^ These modified cells, which exhibit typical pluripotency markers and can differentiate into 3 germ layers, provide a valuable model for studying disease mechanisms of IP and screening therapeutic candidates. However, challenges remain, including the need to correct pathogenic variants in the context of X-chromosome inactivation and mosaicism, particularly in females, and to develop targeted delivery systems for gene therapy. Further research into the genotype–phenotype correlation and the pathophysiological mechanisms of *IKBKG* mutations will be essential to translate these technologies into effective treatments.^4^ Another gene editing therapy that is rising on the horizon is seekRNA, which uses a programmable RNA strand that can directly identify sites for insertion into genetic sequences, thereby reducing errors.^55^ To date, however, there are no studies specifically applying this novel technology to IP.

In the more immediate future, utilization of immunosuppressive agents early on in the disease course to reduce inflammatory markers is being explored.^56^ Tumor necrosis factor-α levels have been shown to be elevated in patients with IP, and recently, both steroids and antitumor necrosis factor-α agents have been used with success.^4^^,^^57^^,^^58^ A recent case report demonstrated control of status epilepticus and resolution of foci of restricted diffusion on brain imaging after the use of oral prednisone in conjunction with levetiracetam.^58^ There are also reports of successful use of infliximab in patients with IP.^59^

## Conclusion

Incontinentia pigmenti is a rare but complex multisystem disorder with potentially blinding retinopathy. Careful ophthalmic screening and surveillance are critical to optimize patients' visual outcomes, but currently, there is no consensus in the literature regarding management of these patients' eye disease. In this review paper, we provide an updated screening, staging, and management guidelines from the ICIP meeting. Key new recommendations include an urgent ophthalmic examination within the first month of diagnosis and evaluation with WFFA in all new patients regardless of the findings of the ophthalmoscopic examination.
